# SARS-CoV-2 RNA Is Readily Detectable at Least 8 Months after Shedding in an Isolation Facility

**DOI:** 10.1128/msphere.00177-22

**Published:** 2022-10-11

**Authors:** David A. Coil, Randi Pechacek, Mo Kaze, Rogelio Zuniga-Montanez, Roque G. Guerrero, Jonathan A. Eisen, Karen Shapiro, Heather N. Bischel

**Affiliations:** a Genome Center, University of California, Davis, Davis, California, USA; b Department of Civil and Environmental Engineering, University of California, Davis, Davis, California, USA; c Department of Medical Microbiology and Immunology, School of Medicine, University of California, Davis, Davis, California, USA; d Department of Evolution and Ecology, University of California, Davis, Davis, California, USA; e Department of Pathology, Microbiology and Immunology, School of Veterinary Medicine, University of California, Davis, Davis, California, USA; Mount Sinai School of Medicine

**Keywords:** COVID-19, RNA, relic RNA, SARS-CoV-2, genome sequencing, surface samples, virus

## Abstract

Environmental monitoring of severe acute respiratory syndrome coronavirus 2 (SARS-CoV-2) for research and public health purposes has grown exponentially throughout the coronavirus disease 2019 (COVID-19) pandemic. Monitoring wastewater for SARS-CoV-2 provides early warning signals of virus spread and information on trends in infections at a community scale. Indoor environmental monitoring (e.g., swabbing of surfaces and air filters) to identify potential outbreaks is less common, and the evidence for its utility is mixed. A significant challenge with surface and air filter monitoring in this context is the concern of “relic RNA,” noninfectious RNA found in the environment that is not from recently deposited virus. Here, we report detection of SARS-CoV-2 RNA on surfaces in an isolation unit (a university dorm room) for up to 8 months after a COVID-19-positive individual vacated the space. Comparison of sequencing results from the same location over two time points indicated the presence of the entire viral genome, and sequence similarity confirmed a single source of the virus. Our findings highlight the need to develop approaches that account for relic RNA in environmental monitoring.

**IMPORTANCE** Environmental monitoring of SARS-CoV-2 is rapidly becoming a key tool in infectious disease research and public health surveillance. Such monitoring offers a complementary and sometimes novel perspective on population-level incidence dynamics relative to that of clinical studies by potentially allowing earlier, broader, more affordable, less biased, and less invasive identification. Environmental monitoring can assist public health officials and others when deploying resources to areas of need and provides information on changes in the pandemic over time. Environmental surveillance of the genetic material of infectious agents (RNA and DNA) in wastewater became widely applied during the COVID-19 pandemic. There has been less research on other types of environmental samples, such as surfaces, which could be used to indicate that someone in a particular space was shedding virus. One challenge with surface surveillance is that the noninfectious genetic material from a pathogen (e.g., RNA from SARS-CoV-2) may be detected in the environment long after an infected individual has left the space. This study aimed to determine how long SARS-CoV-2 RNA could be detected in a room after a COVID-positive person had been housed there.

## OBSERVATION

The ability to detect severe acute respiratory syndrome coronavirus 2 (SARS-CoV-2) in the environment has proven useful in a number of settings during the coronavirus disease 2019 (COVID-19) pandemic. Most well-known is the potential early detection of viral RNA in wastewater relative to that from clinical testing (1–4). Other work has focused on surface and/or air filter sampling (5, 6). Interpretation of results from surface and/or air filter samples is challenging due to the presence of “relic RNA,” i.e., RNA present in the environment long after shedding has occurred (7, 8). SARS-CoV-2 RNA has been detected in the environment for up to 2 months after shedding (9). In this study, we sampled a university dormitory room that had been used for isolation of a patient following a positive SARS-CoV-2 test. We systematically tested surfaces for SARS-CoV-2 for 8 months after this room was vacated. SARS-CoV-2 RNA was quantified through reverse transcription-quantitative PCR (RT-qPCR), and a subset of positive samples was sequenced.

### Results and discussion.

**FIG 1:**
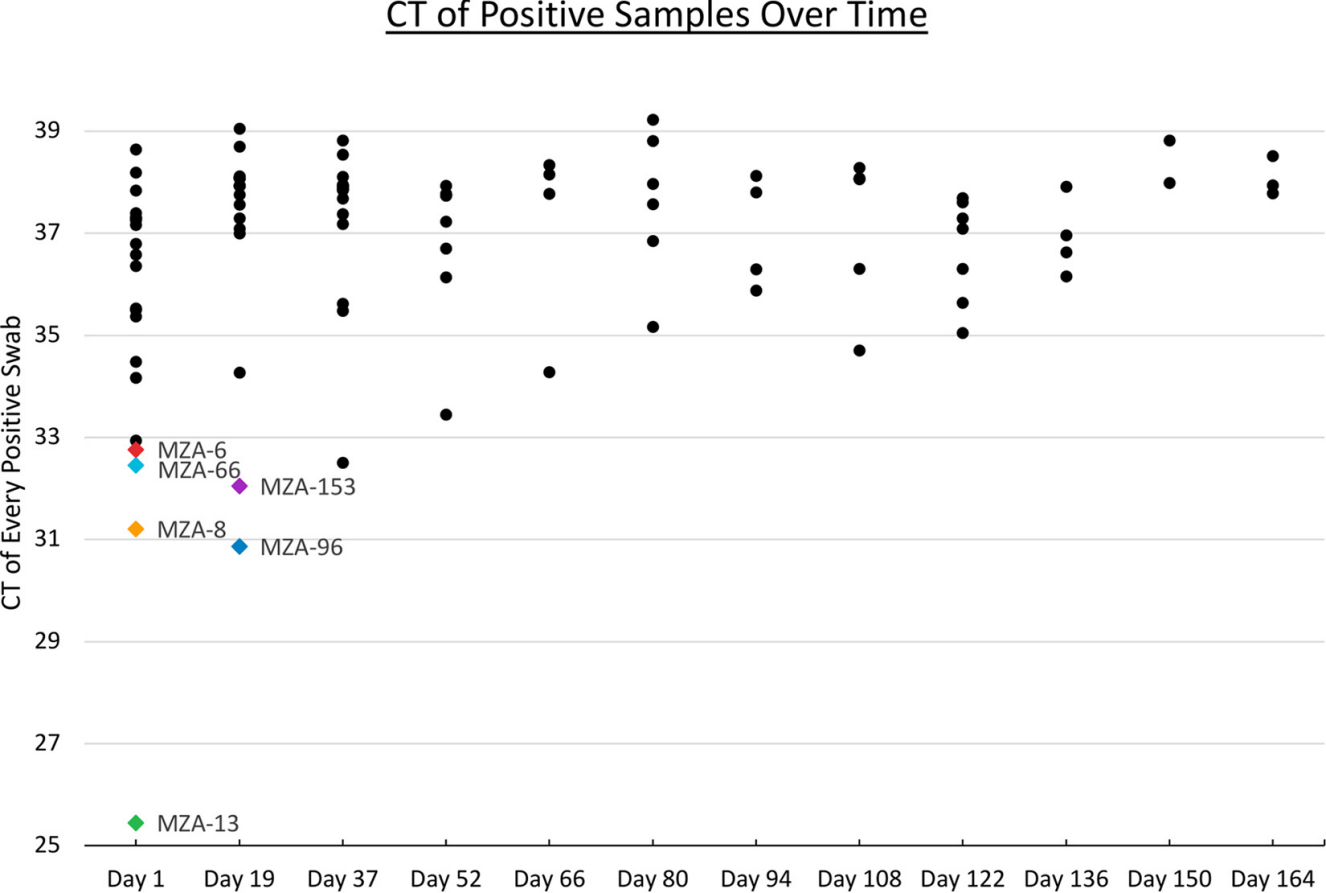
Cycle threshold (*C_T_*) values for positive samples over time. Each dot represents a unique sample. Sampling was also conducted on 8 December 2021 (day 178), but no positives were detected at that time. The colored dots represent samples for which a genome sequence was generated (see the key in Fig. 2).

SARS-CoV-2 RNA was detectable on surfaces and air filters for 8 months after the room had been occupied (Fig. 1). The frequency of positive samples per sampling episode declined over time, from 70% in the first sampling event to 0% in the last sampling event. The average threshold cycle (*C_T_*) per sampling session increased from just over 35 at the start to 38 over this period. While these data indicate that the RNA does degrade through time, detection remained possible for much longer than we expected.

**FIG 2:**
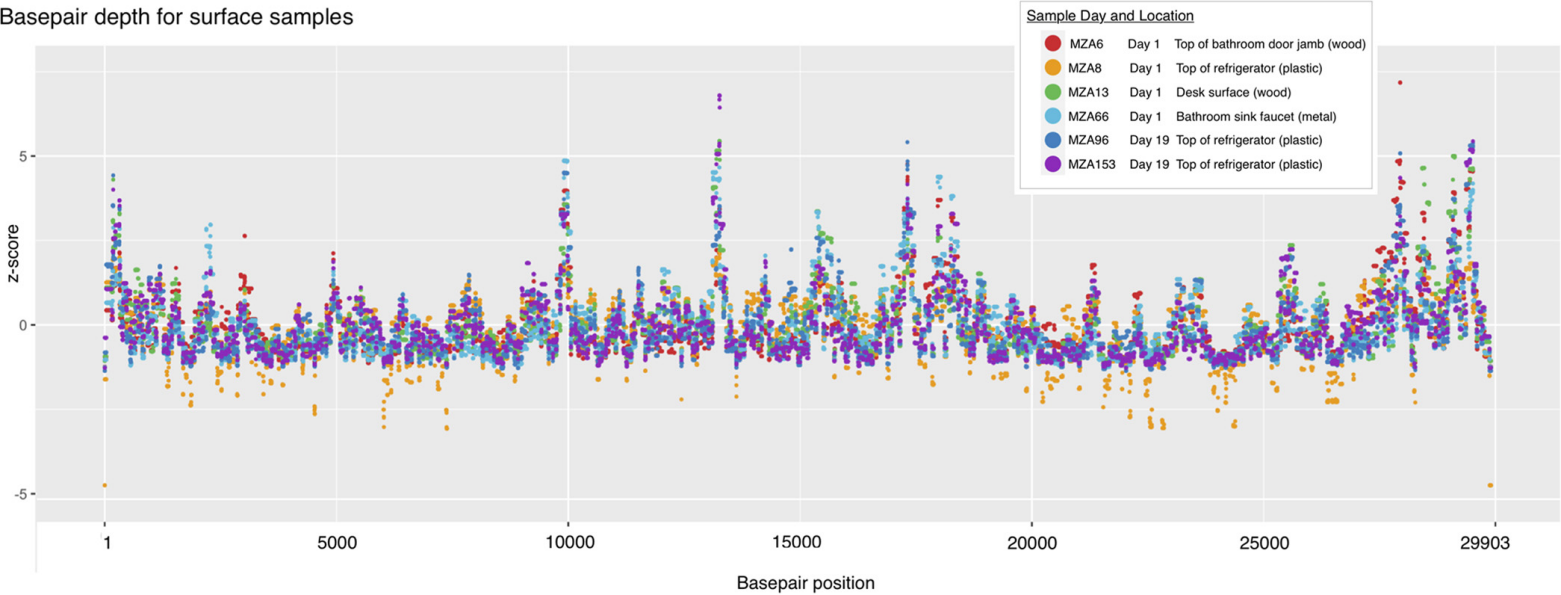
Normalized base-pair depth of coverage along the SARS-CoV-2 genome for six high-quality samples from two time points and four sampling surface types.

To obtain additional insights into RNA degradation, we examined the depth of sequencing coverage across the SARS-CoV-2 genome (Fig. 2). Since many factors can cause variation in sequencing runs, depth of coverage was normalized within each sample. As seen with fresh SARS-CoV-2 samples, there was variation in depth of coverage across the genome (10, 11). Overall, the depths of coverage for the samples paralleled each other and demonstrated that complete SARS-CoV-2 genomes could be assembled from surface samples collected at least 74 days after the room had been occupied. There were some differences in genome coverage that could be indicative of degradation over time. For example, there were regions of the genome where the normalized coverage differed between samples and was higher for day 1 samples than day 19 samples. We consider that further evaluation of genome coverage is worth pursuing as a potential way to estimate the age of RNA in a sample.

In a study of SARS-CoV-2 RNA on high-touch outdoor surfaces, weekly sample positive rates most closely corresponded with COVID-19 cases and offered an early warning of community transmission (12). Our results from an indoor, quiescent setting showed that there is potential for a long time lag between RNA deposition and detection. Previous related work found that detection of relic RNA is dependent on the surface type and highlighted the importance of cleaning surfaces between collections in order to understand the dynamics of deposition (13).

Our results highlight the importance of considering the stability of SARS-CoV-2 RNA over time in the context of surface-based virus surveillance. New methods to bracket the “age” of RNA in the environment would provide immense value in assessing whether detected RNA is from recent deposition or represents relic RNA. Based on the lack of demonstrated fomite transmission of SARS-CoV-2 to date, it is unlikely any detectable RNA in the environment is infectious. However, the ability to take action in response to environmental data depends on the time since the viable virus was shed by infected individuals in a defined space.

### Materials and methods: study location.

All sampling took place in a single isolation unit at UC Davis that was occupied from 18 to 20 April 2021 by a COVID-19-positive student. Sampling began 55 days after the room had been last occupied and the room was not used for anything else. Sampling took place approximately every 2 weeks until mid-December 2021. A total of 264 surface samples were collected during the study duration. For each sampling episode, 14 to 30 samples were collected from a variety of surfaces. After collection of surface samples on each the first six sampling episodes, vigorous shuffling on the carpet and movement of numerous surfaces was performed throughout the unit. A portable HEPA air filtration unit was then operated for 1 h. The HEPA unit (mesh) was sampled before and after operation.

### Sample collection.

Environmental sampling was conducted as previously described (7). Briefly, samples were collected using nylon fiber oral swabs with an ABS handle (Miraclean Technology Co. Ltd., China) that were premoistened in DNA/RNA Shield (Zymo Research, USA). An area of 10 cm by 10 cm (or equivalent) was swabbed for surface samples, and a similar process was used to swab the exterior surface of the HEPA filter (~823 cm^2^) in a portable filtration unit. The same 10-cm by 10-cm area was swabbed at every sampling time point until day 37. Afterwards, a different 10-cm by 10-cm area was swabbed every time for most locations.

### RNA extraction and RT-qPCR.

RNA extraction and RT-qPCR were performed as previously described (7). Samples were extracted using the MagMAX Microbiome Ultra nucleic acid isolation kit (Applied Biosystems, USA) with a KingFisher Flex automated purification system (Thermo Fisher Scientific, USA). The MagMAX_Microbiome_Stool_Flex.bdz nucleic acid isolation protocol (Applied Biosystems, USA) was utilized, with modifications. In brief, the sample lysis step was not conducted, as lysis was achieved through the use of DNA/RNA Shield and vortexing. All extracts were analyzed by RT-qPCR targeting the spike glycoprotein (S) gene of SARS-CoV-2 (14) using the Luna universal probe one-step RT-qPCR kit (New England Biolabs Inc., USA).

### Sequencing.

Eight samples from the room were chosen for sequencing alongside other samples from related projects. The samples across projects were chosen to represent a variety of *C_T_* values and surface types to calibrate future sequencing efforts. Ten microliters of extracted nucleic acid from each sample was converted to cDNA by using the LunaScript RT SuperMix kit (New England Biolabs, Ipswich, MA) in a 20-μL reaction mix, which was incubated at 25°C for 2 min followed by 55°C for 10 min and heat inactivated at 95°C for 1 min. Subsequently, 10 μL of this first-strand cDNA was used as input for amplification of the SARS-CoV-2 viral genome, using the xGen SARS-CoV-2 amplicon panel (IDT, Coralville, IA), which consisted of 345 amplicons covering 99.7% of the SARS-CoV-2 Wuhan-Hu-1 strain (NC_045512.2). The workflow uses a single tube of tiled primer pairs that result in an average amplicon size of 150 bp. The amplicon libraries were generated using the low viral load workflow (*C_T_* > 20), which involves two PCR rounds, a multiplex PCR (4 + 24 cycles) and the indexing PCR (5 cycles) to generate sequence-ready libraries. Libraries were barcoded with 8-bp unique dual indices during the indexing PCR. Equimolar libraries were pooled and quantified by qPCR with the KAPA library quantification kit (Roche, Basel, Switzerland). The pooled library was sequenced on one lane of an Illumina Mid Output NextSeq 500 system (Illumina, San Diego, CA) with paired-end 150-bp reads. Two of the eight samples from the room did not generate sufficient library material for sequencing.

### Bioinformatics.

Sequence reads for the remaining 6 samples were aligned to the SARS-CoV-2 reference genome, NCBI accession number NC_045512, by using bowtie2 v2.4.5 (15). Samtools v1.3.1 (16) was used to index, sort, and filter by quality (alignments with MAPQ of <20 were skipped) and to quantify unique reads for each genome position. Reads were used to generate a consensus sequence from all 6 samples. Base-pair depth per each genome position was normalized to the mean and visualized with ggplot2 v3.3.4 (17) and Preview.app.

### Data availability.

Raw and processed sequence data from this study have been uploaded onto Dryad and are publicly accessible and available for research use at: http://doi.org/10.25338/B8GP94.

## References

[1] Zhu Y, Oishi W, Maruo C, Saito M, Chen R, Kitajima M, Sano D. 2021. Early warning of COVID-19 via wastewater-based epidemiology: potential and bottlenecks. Sci Total Environ 767:145124. doi:10.1016/j.scitotenv.2021.145124. 33548842 PMC7825884

[2] Hrudey SE, Conant B. 2022. The devil is in the details: emerging insights on the relevance of wastewater surveillance for SARS-CoV-2 to public health. J Water Health 20:246–270. doi:10.2166/wh.2021.186. 35100171

[3] Bibby K, Bivins A, Wu Z, North D. 2021. Making waves: plausible lead time for wastewater based epidemiology as an early warning system for COVID-19. Water Res 202:117438. doi:10.1016/j.watres.2021.117438. 34333296 PMC8274973

[4] Kirby AE, Welsh RM, Marsh ZA, Yu AT, Vugia DJ, Boehm AB, Wolfe MK, White BJ, Matzinger SR, Wheeler A, Bankers L, Andresen K, Salatas C, Gregory DA, Johnson MC, Trujillo M, Kannoly S, Smyth DS, Dennehy JJ, Sapoval N, Ensor K, Treangen T, Stadler LB, Hopkins L, New York City Department of Environmental Protection. 2022. Notes from the field: early evidence of the SARS-CoV-2 B.1.1.529 (omicron) variant in community wastewater—United States, November–December 2021. MMWR Morb Mortal Wkly Rep 71:103–105. doi:10.15585/mmwr.mm7103a5. 35051130 PMC8774157

[5] Borges JT, Nakada LYK, Maniero MG, Guimarães JR. 2021. SARS-CoV-2: a systematic review of indoor air sampling for virus detection. Environ Sci Pollut Res Int 28:40460–40473. doi:10.1007/s11356-021-13001-w. 33630259 PMC7905194

[6] Coil DA, Albertson T, Banerjee S, Brennan G, Campbell AJ, Cohen SH, Dandekar S, Díaz-Muñoz SL, Eisen JA, Goldstein T, Jose IR, Juarez M, Robinson BA, Rothenburg S, Sandrock C, Stoian AMM, Tompkins DG, Tremeau-Bravard A, Haczku A. 2021. SARS-CoV-2 detection and genomic sequencing from hospital surface samples collected at UC Davis. PLoS One 16:e0253578. doi:10.1371/journal.pone.0253578. 34166421 PMC8224861

[7] Zuniga-Montanez R, Coil DA, Eisen JA, Pechacek R, Guerrero RG, Kim M, Shapiro K, Bischel HN. 2022. The challenge of SARS-CoV-2 environmental monitoring in schools using floors and portable HEPA filtration units: fresh or relic RNA? PLos One 17:e0267212. doi:10.1371/journal.pone.0267212. 35452479 PMC9032406

[8] Renninger N, Nastasi N, Bope A, Cochran SJ, Haines SR, Balasubrahmaniam N, Stuart K, Bivins A, Bibby K, Hull NM, Dannemiller KC. 2021. Indoor dust as a matrix for surveillance of COVID-19. mSystems 6. doi:10.1128/mSystems.01350-20. PMC8547012 33850045

[9] Maestre JP, Jarma D, Yu J-RF, Siegel JA, Horner SD, Kinney KA. 2021. Distribution of SARS-CoV-2 RNA signal in a home with COVID-19 positive occupants. Sci Total Environ 778:146201. doi:10.1016/j.scitotenv.2021.146201. 34030356 PMC7942153

[10] Addetia A, Lin MJ, Peddu V, Roychoudhury P, Jerome KR, Greninger AL. 2020. Sensitive recovery of complete SARS-CoV-2 genomes from clinical samples by use of Swift Biosciences’ SARS-CoV-2 multiplex amplicon sequencing panel. J Clin Microbiol 59. doi:10.1128/JCM.02226-20. PMC7771467 33046529

[11] Orf GS, Forberg K, Meyer TV, Mowerman I, Mohaimani A, Faron ML, Jennings C, Landay AL, Goldstein DY, Fox AS, Berg MG, Cloherty GA. 2021. SNP and phylogenetic characterization of low viral load SARS-CoV-2 specimens by target enrichment. FrontVirol 1. doi:10.3389/fviro.2021.765974.

[12] Harvey AP, Fuhrmeister ER, Cantrell ME, Pitol AK, Swarthout JM, Powers JE, Nadimpalli ML, Julian TR, Pickering AJ. 2021. Longitudinal monitoring of SARS-CoV-2 RNA on high-touch surfaces in a community setting. Environ Sci Technol Lett 8:168–175. doi:10.1021/acs.estlett.0c00875. 34192125 PMC7927285

[13] Salido RA, Cantú VJ, Clark AE, Leibel SL, Foroughishafiei A, Saha A, Hakim A, Nouri A, Lastrella AL, Castro-Martínez A, Plascencia A, Kapadia BK, Xia B, Ruiz CA, Marotz CA, Maunder D, Lawrence ES, Smoot EW, Eisner E, Crescini ES, Kohn L, Franco Vargas L, Chacón M, Betty M, Machnicki M, Wu MY, Baer NA, Belda-Ferre P, De Hoff P, Seaver P, Ostrander RT, Tsai R, Sathe S, Aigner S, Morgan SC, Ngo TT, Barber T, Cheung W, Carlin AF, Yeo GW, Laurent LC, Fielding-Miller R, Knight R. 2021. Analysis of SARS-CoV-2 RNA persistence across indoor surface materials reveals best practices for environmental monitoring programs. mSystems 6:e0113621. doi:10.1128/mSystems.01136-21. 34726486 PMC8562474

[14] Chan JF-W, Yuan S, Kok K-H, To KK-W, Chu H, Yang J, Xing F, Liu J, Yip CC-Y, Poon RW-S, Tsoi H-W, Lo SK-F, Chan K-H, Poon VK-M, Chan W-M, Ip JD, Cai J-P, Cheng VC-C, Chen H, Hui CK-M, Yuen K-Y. 2020. A familial cluster of pneumonia associated with the 2019 novel coronavirus indicating person-to-person transmission: a study of a family cluster. Lancet 395:514–523. doi:10.1016/S0140-6736(20)30154-9. 31986261 PMC7159286

[15] Langmead B, Salzberg SL. 2012. Fast gapped-read alignment with Bowtie 2. Nat Methods 9:357–359. doi:10.1038/nmeth.1923. 22388286 PMC3322381

[16] Danecek P, Bonfield JK, Liddle J, Marshall J, Ohan V, Pollard MO, Whitwham A, Keane T, McCarthy SA, Davies RM, Li H. 2021. Twelve years of SAMtools and BCFtools. Gigascience 10. doi:10.1093/gigascience/giab008. PMC7931819 33590861

[17] Chang W, Pedersen H. 2022. ggplot2: create elegant data visualisations using the grammar of graphics. R package version. https://github.com/tidyverse/ggplot2.

